# Expandable grid, a simple, readily available and precise technique for intraoperative stereotactic real-time imaging

**DOI:** 10.1007/s00701-025-06505-8

**Published:** 2025-04-02

**Authors:** Dirar Aldabek, Andreas Hodul, François Alesch

**Affiliations:** 1 Department of Neurosurgery, Heinrich-Braun-Klinikum, Karl-Keil-Straße 35, 08060 Zwickau, Germany; 2 Center for Medical Physics and Biomedical Engineering, Medical University of Vienna, Spitalgasse 23, 1090 Vienna, Austria; 3 Department of Neurosurgery, Medical University of Vienna, Spitalgasse 23, 1090 Vienna, Austria

**Keywords:** Fluoroscopy, C-Arm, Stereotaxy, DBS, Intraoperative imaging

## Abstract

**Introduction:**

Pre- and postoperative imaging constitutes a firm brick in planning and steering accurate stereotactic procedures. The availability of intraoperative control measures, e.g., CT, MRI, and microelectrode recording (MER), is often limited to a minority of centers. Our approach utilizes fluoroscopy for target planning and coordinates validation as control.

**Methods:**

This technique was primarily conceived for the RM (Riechert Mundinger) stereotactic system, but it also applies to the ZD (Zamorano—Dujovny) system. In the present study, we shifted the zero of the Z-value (axis of the patient) to + 60 mm. This corresponds to the center of the Angio/X-ray localizing plates. By assigning a radiopaque marker to the center of each plate, aligning these centers produced orthogonal and non-distorted stereotactic space. In this space, the magnification variable matters to us the most. Using available viewer software, we printed a millimetric grid on translucent foils with the corresponding magnification factor, which can easily be superimposed on the fluoroscopic image. This allows the precise validation of the coordinates of points of interest, including typical stereotactic landmarks. This technique can be used in both views, AP and lateral.

**Results:**

We have validated this technique under non-clinical (phantom) conditions and with intraoperative images obtained during routine stereotactic procedures. The latter were acquired using our classical stereotactic fixedly-mounted X-ray system. We found identical results, with an accuracy margin of error lower than 1 mm.

**Conclusion:**

This simple geometrical adaptation proved to be an accurate, accessible, mobile, and manageable technique providing immediate access to stereotactic coordinates during surgery. The accuracy proved to be non-inferior to other more complex and time-consuming imaging modalities.

## Introduction

During the early stages of functional stereotaxy, non-tomography-based X-ray imaging was the mainstay for planning and targeting, as highlighted by Talairach in 1957 [5]. This combined with angiography and brain atlases, formed the basis for stereotactic planning [3]. Yet, obtaining accurate stereotactic X-ray representations required extensive structural modifications of the operative field.

**Fig. 1:**
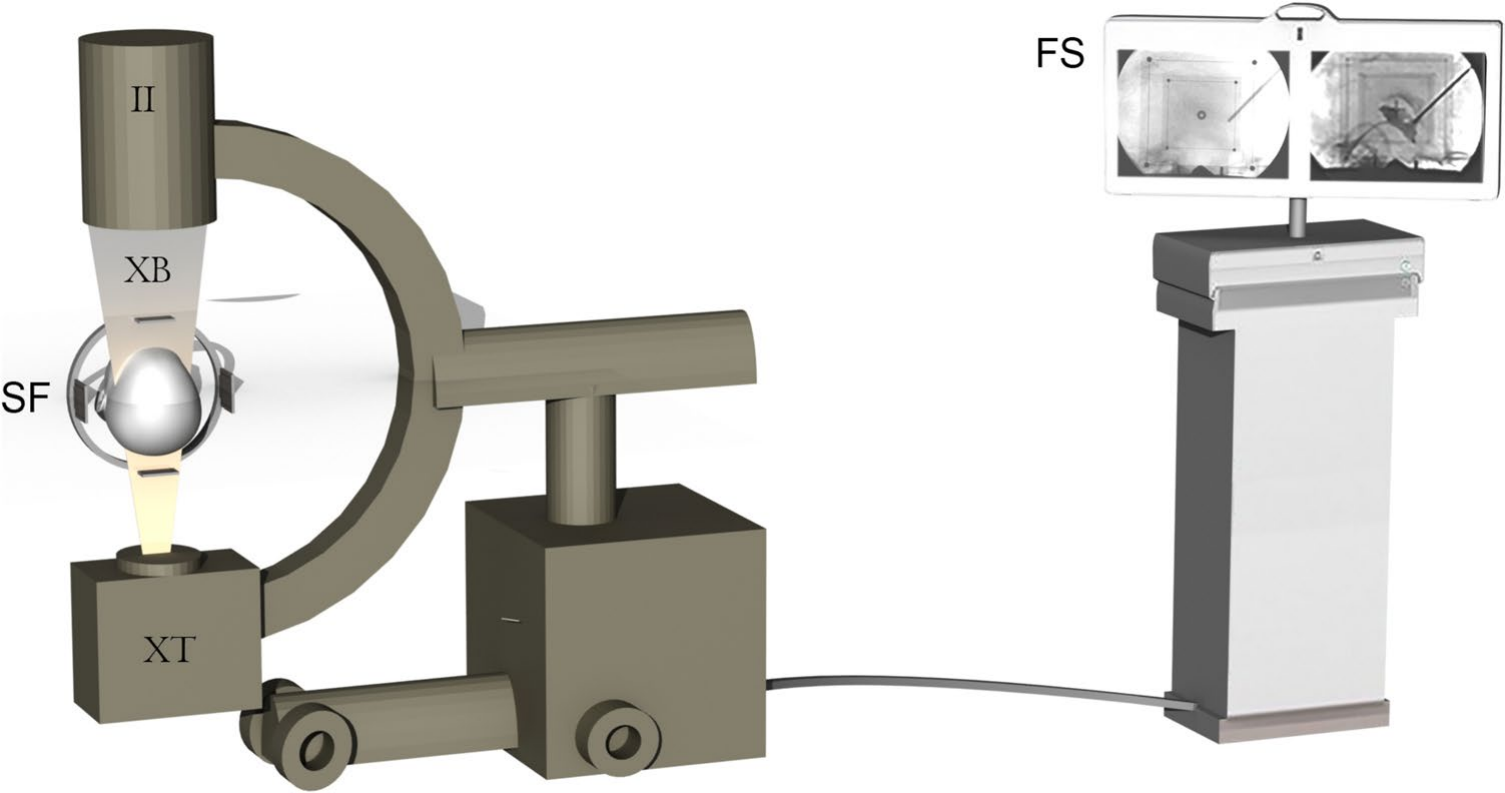
(II) Image intensifier, (XT) X-Ray tube, (XB) X-Ray beam, (SF) Stereotactic frame with fitted localizer plates, (FS) Flat-screen to visualize obtained images

The advent of tomography-based techniques, starting with computerized tomography and later magnetic resonance imaging, significantly diminished the importance of traditional intraoperative X-ray imaging [2]. The broad implementation of intraoperative Microelectrode recording provided essential information for pinpointing stereotactic target points [1]. The use of fluoroscopy in stereotactic neurosurgery was limited to a few applications, including cyst punctures and biopsies. Yet, fluoroscopic images cannot be calibrated, rendering their use for precisely calculating or mathematically delineating stereotactic target points unreliable. This study introduces a method to calibrate and reference fluoroscopy images for use in stereotactic spaces (Fig. 1).

## Methods/Design

**Fig. 2:**
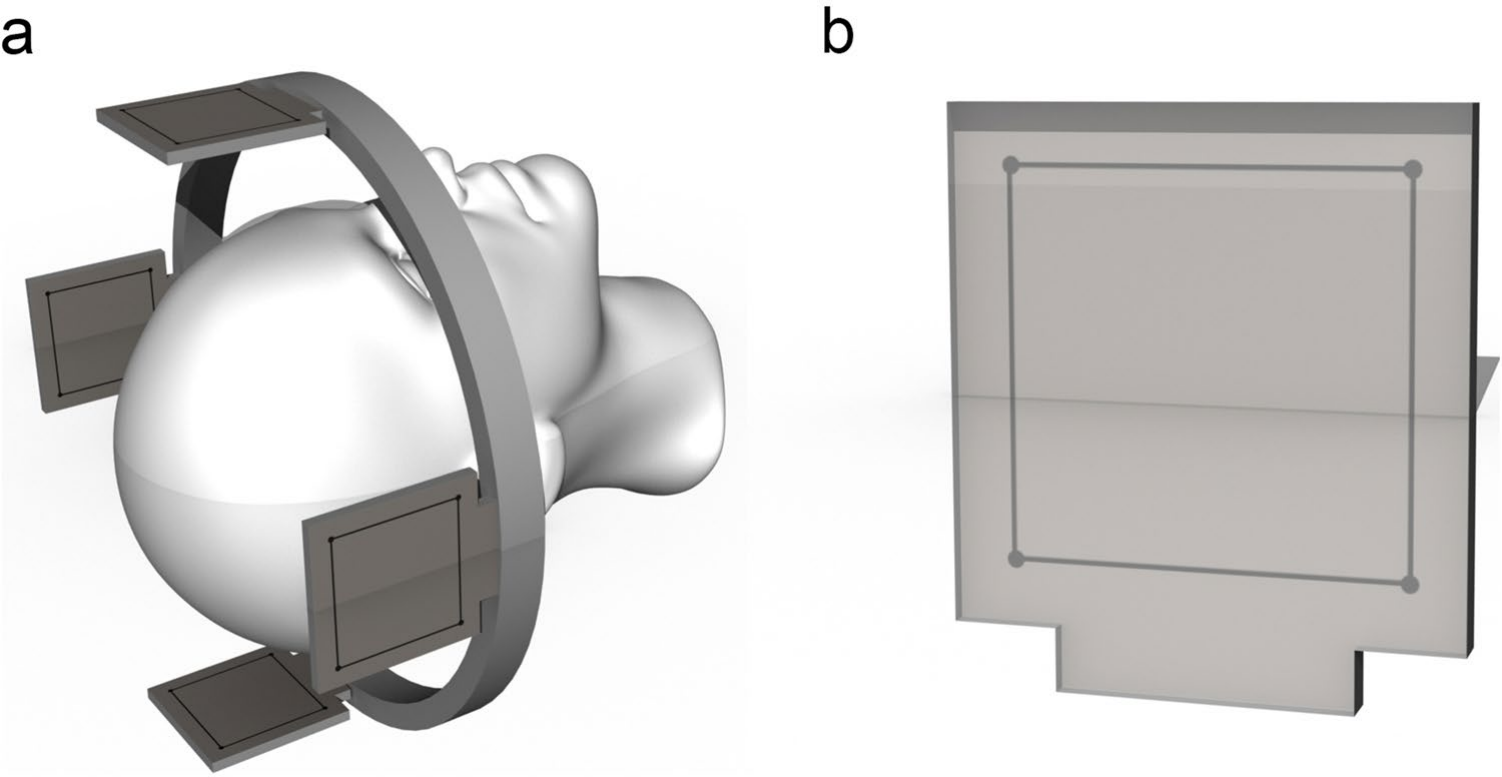
**a** Stereotactic ring with fitted four localizer plates. **b** shows the geometry of each single localizer plate with the characteristic square 60 × 60 mm radio-opaque marks

**Fig. 3:**
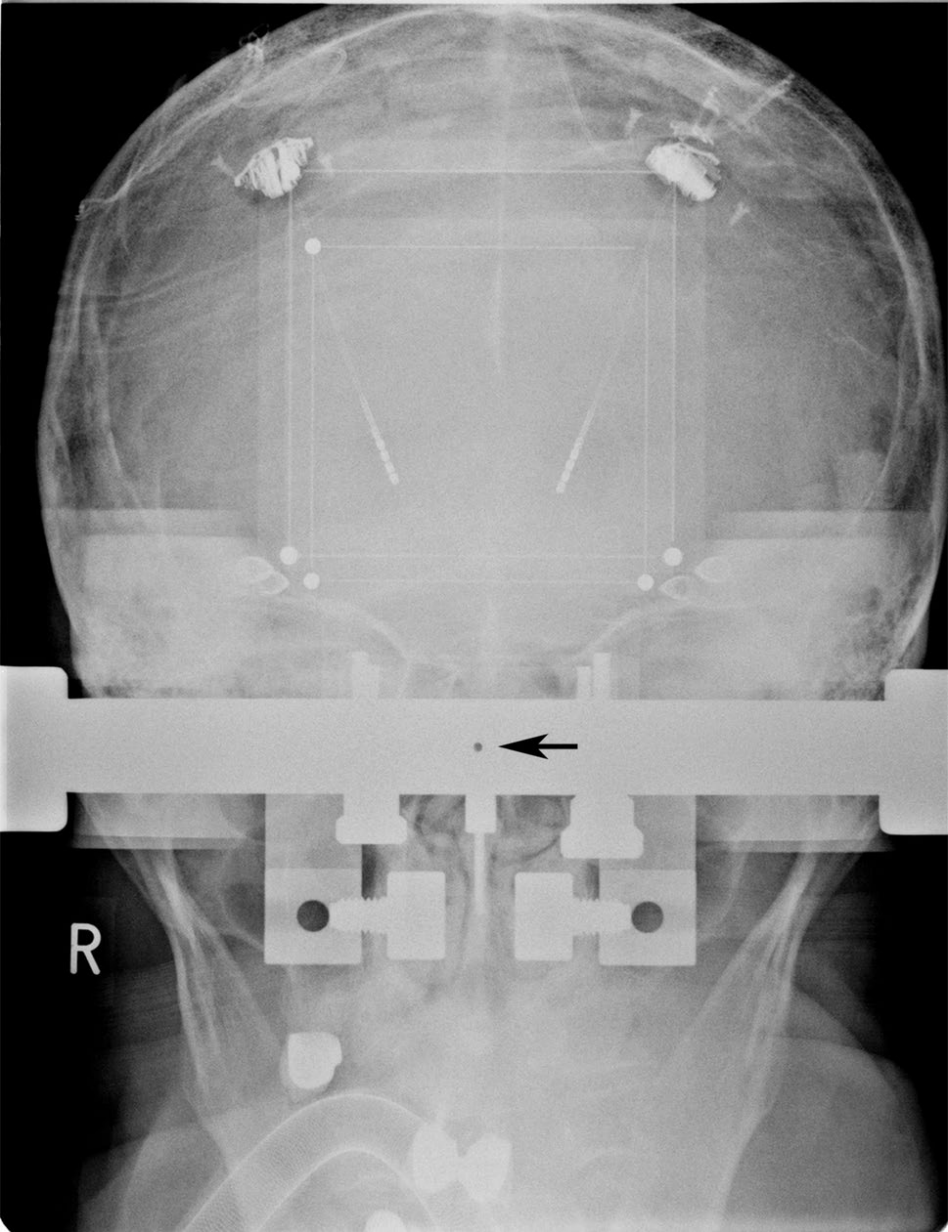
AP image obtained through a fixedly-mounted x-ray setting. The arrow points to the center of the image, where the X-ray beam is orthogonal

**Fig. 4:**
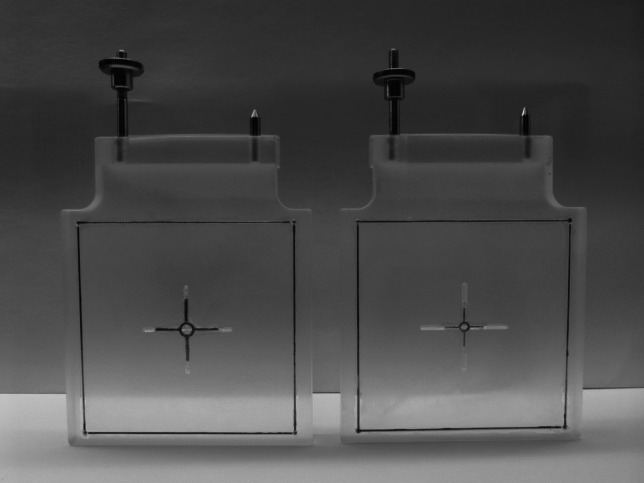
The angio-plates used in conventional fluoroscopic procedures are modified by adding a ring and a cross to the center of each plate. The dimensions of the markers on every two opposing plates respect the different magnification factors of each plate. The plate closer to the source of the x-ray beam has smaller markers (relatively significant magnification), while the other plate (left) is marked with larger ring and cross (relatively minimal magnification)

**Fig. 5:**
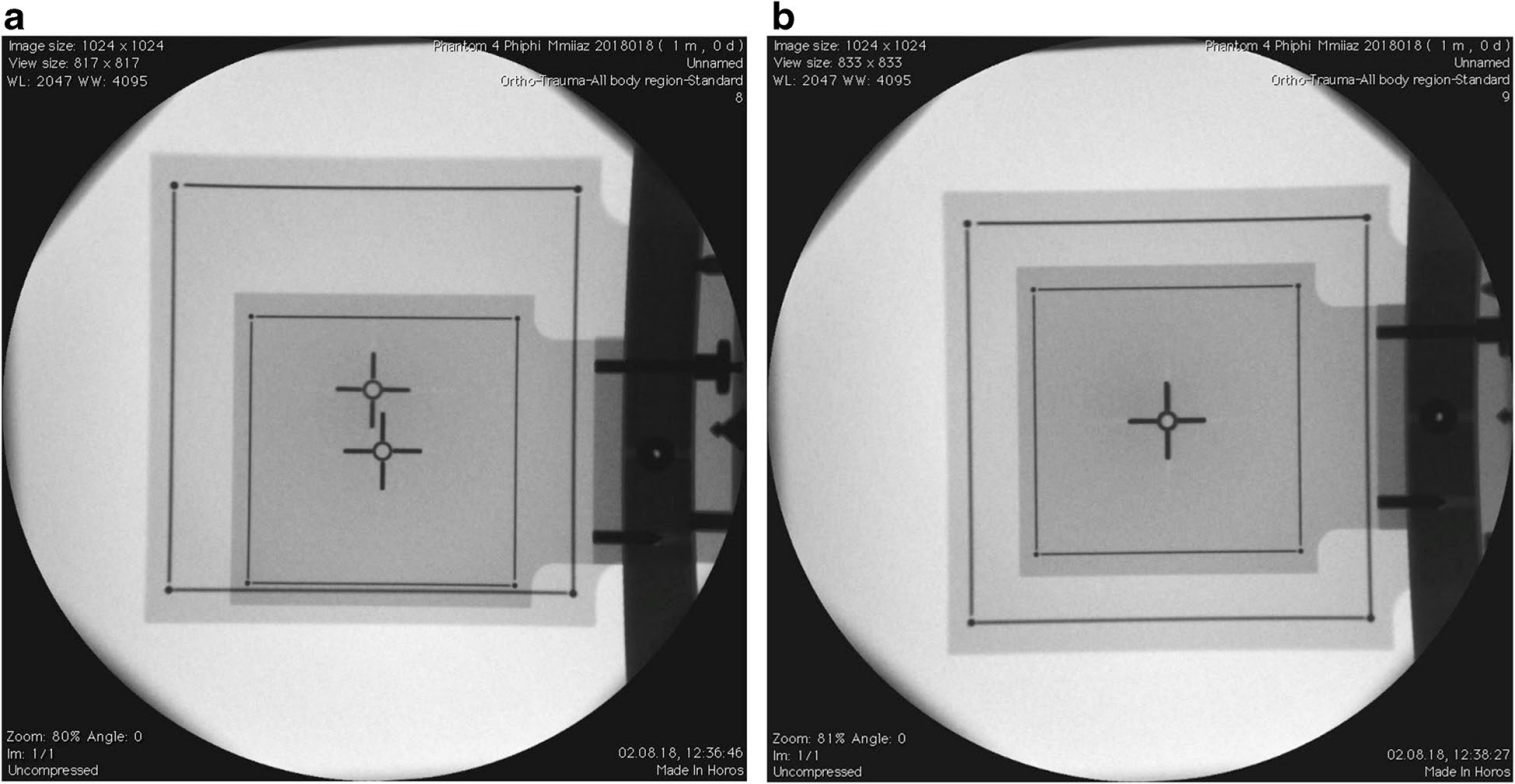
**a** The image shows the process of superimposition. **(b)** The image shows the superimposed plates

Our technique is primarily conceived for the Riechert-Mundinger (RM) stereotactic system but also applies to the Zamorano-Dujovny (ZD) and Stereotactic Ultralight System [4]. All use the same referential geometry and share the same localizers (Fig. 2). The standard zero-point for all three coordinates (x, y, z) of the RM referential space is the center of the frame (Fig. 3). In addition to the CT- or MRI-localizers typically used to define coordinates in CT and MRI respectively, the RM system also adapts to the Angio/X-ray localizing plates (inomed Medizintechnik GmbH, item no.: 809000), whose purpose is to determine the coordinates of a target point using classical angiographic or even digital subtraction (DSA) images. Each acrylic plate has four radiopaque dots that allow calibrating and referencing classical radiographic pictures using dedicated software. We modified the plates by adding crosshairs in their center to fulfill our purpose (Fig. 4). Crosshairs at the center of the plates facilitate the superposition of the lateral or anterior/posterior plates in lateral and AP views during fluoroscopy. Aligning each opposing plate pair ensures a perfect orthogonal view and hence produces a projection image of a non-distorted stereotactic space (Fig. 5). This implies that the center of our radiological stereotactic space should correspond to the center of the crosshairs, that is z = 60 mm. This modification does not involve the X and Y coordinates. This has several advantages: in a typical setup for functional neurosurgery using the RM system, the anterior and the posterior commissure are located near this center. Image distortion, therefore, is negligible at that level. Furthermore, shifting the reference point 60 mm distal to the metallic ring (z = 0) avoids the substantial radioopacity caused by the ring, thereby improving the overall clarity and precision of the radiographic imaging.

**Fig. 6:**
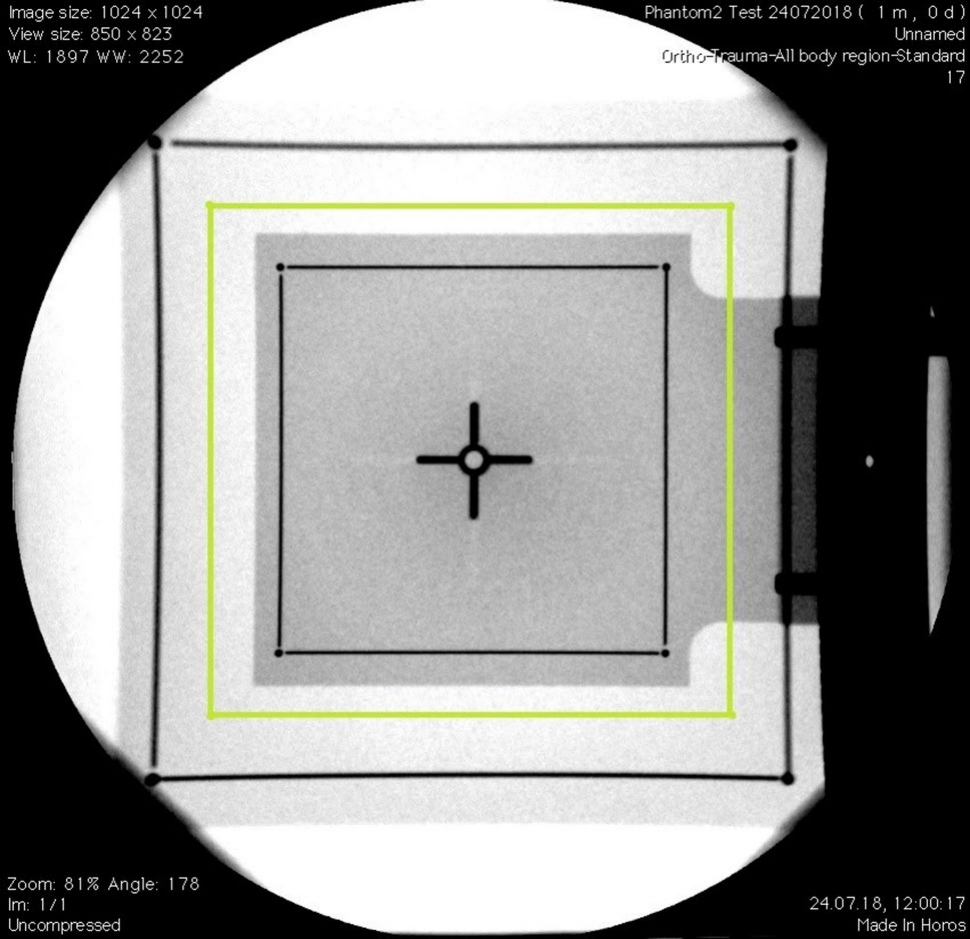
Lateral view showing the two localizer plates. The imaginary midsagittal plate is drawn and marked in green

The produced images vary in size for every imaging session depending on the distance between the source of the X-ray beam and the object. We calculate the magnification of the produced images upon calibrating the crosshairs at the beginning of each session. Given the true physical distance of 360 mm between every two opposing localizer plates, these project with two different magnifications on the screen. Typically, key stereotactic targets—such as the subthalamic nucleus (STN), the ventral intermediate nucleus (Vim), and the zona incerta (Zi)—are located midway between the two localizers. Consequently, determining the magnification factor based on the projected image of an imaginary mid-localizer provides an accurate representation of the actual magnification of the working plane. The on-the-image-produced dimension of both squares allows us to calculate the dimensions of the aforementioned imaginary localizer by obtaining the mean dimensions of both projected plates (Fig. 6).

$$S_{mean}=\frac{length\;of\;one\;proximal\;side+length\;of\;one\;distal\;side}2$$

$S_{mean}$: The mean length of one side of the imaginary localizer.

The magnification factor of this midsagittal plate is calculated by dividing the length of one of its sides by the true length of each plate arm, which is 60 mm.

$$\text{Magnification }(\text{M})=\frac{{S}_{mean}}{60}$$

**Fig. 7:**
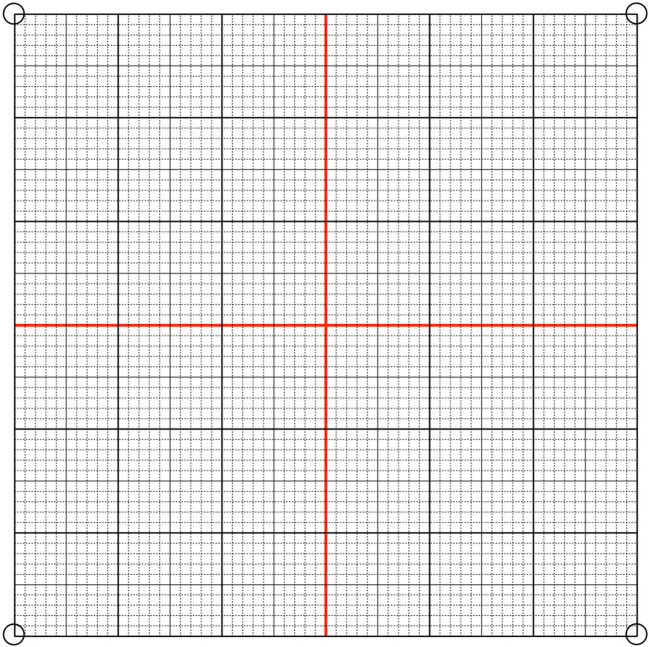
Showing 60*60 millimetric grid

This calculated magnification factor is then employed to print a magnified 60 × 60 millimetric math grid (Fig. 7) on a translucent sheet. This sheet is superimposed on the produced image on the screen and is used to define various targets and confirm immediately their coordinates.

**Fig. 8:**
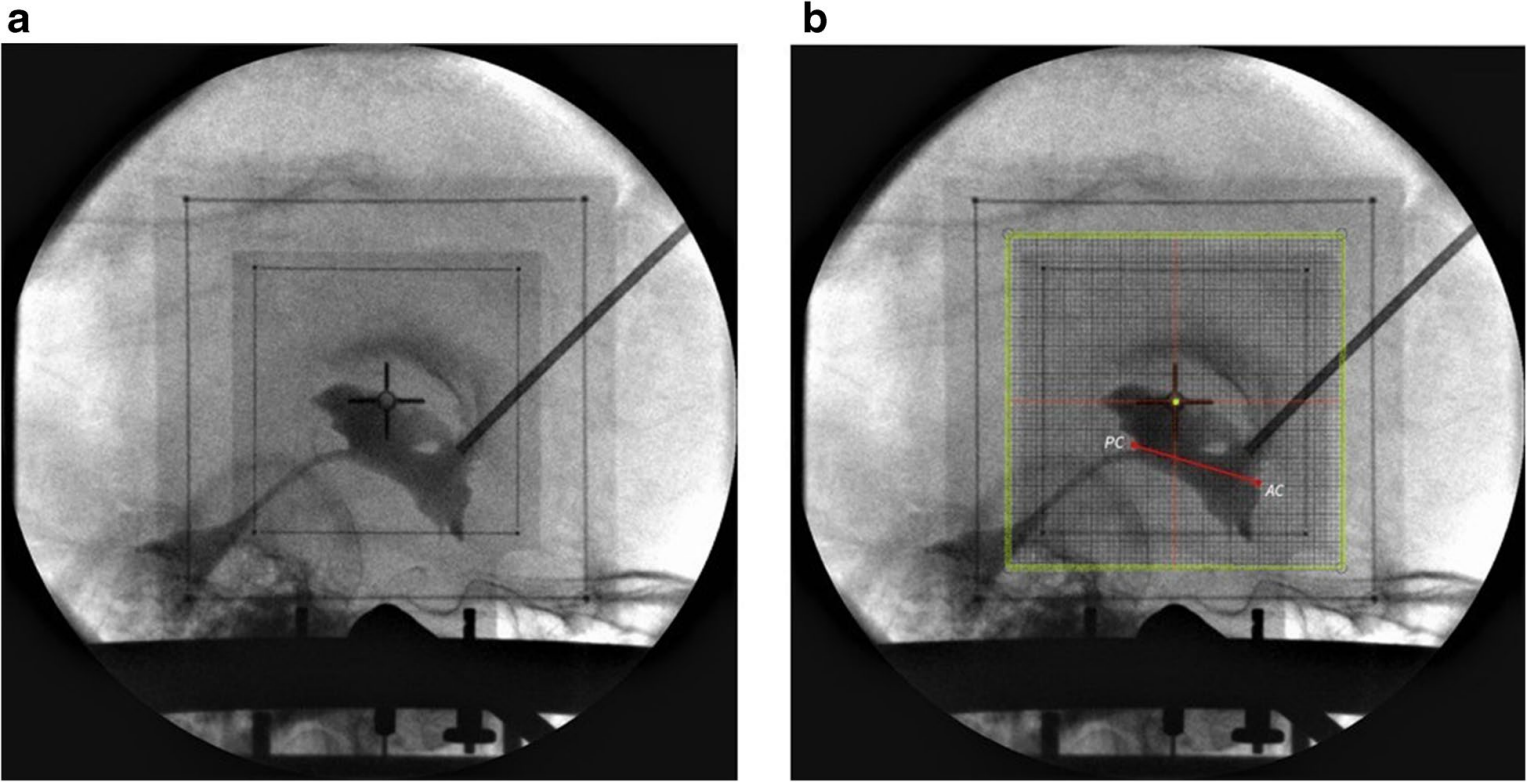
**a** Ventriculography in lateral view. The system is centralized. **b** The green dot was drawn to point to the center of the system (x NA, y 0, z + 60). After the grid was superimposed over the image, the coordinates of the AC-PC line were calculated

The use of intraoperative C-arm can be broadened through the classic visualization of the third ventricle. We achieve this by injecting 1–2 ml of contrast agent in the foramen of Monro stereotactically. Incorporating the visualization of key anatomical landmarks—including the anterior commissure, posterior commissure, the massa intermedia (if present), and the floor of the third ventricle—helps ensure the anatomical accuracy of the target selection, stereotactic precision as well as reliability (Fig. 8).

**Fig. 9:**
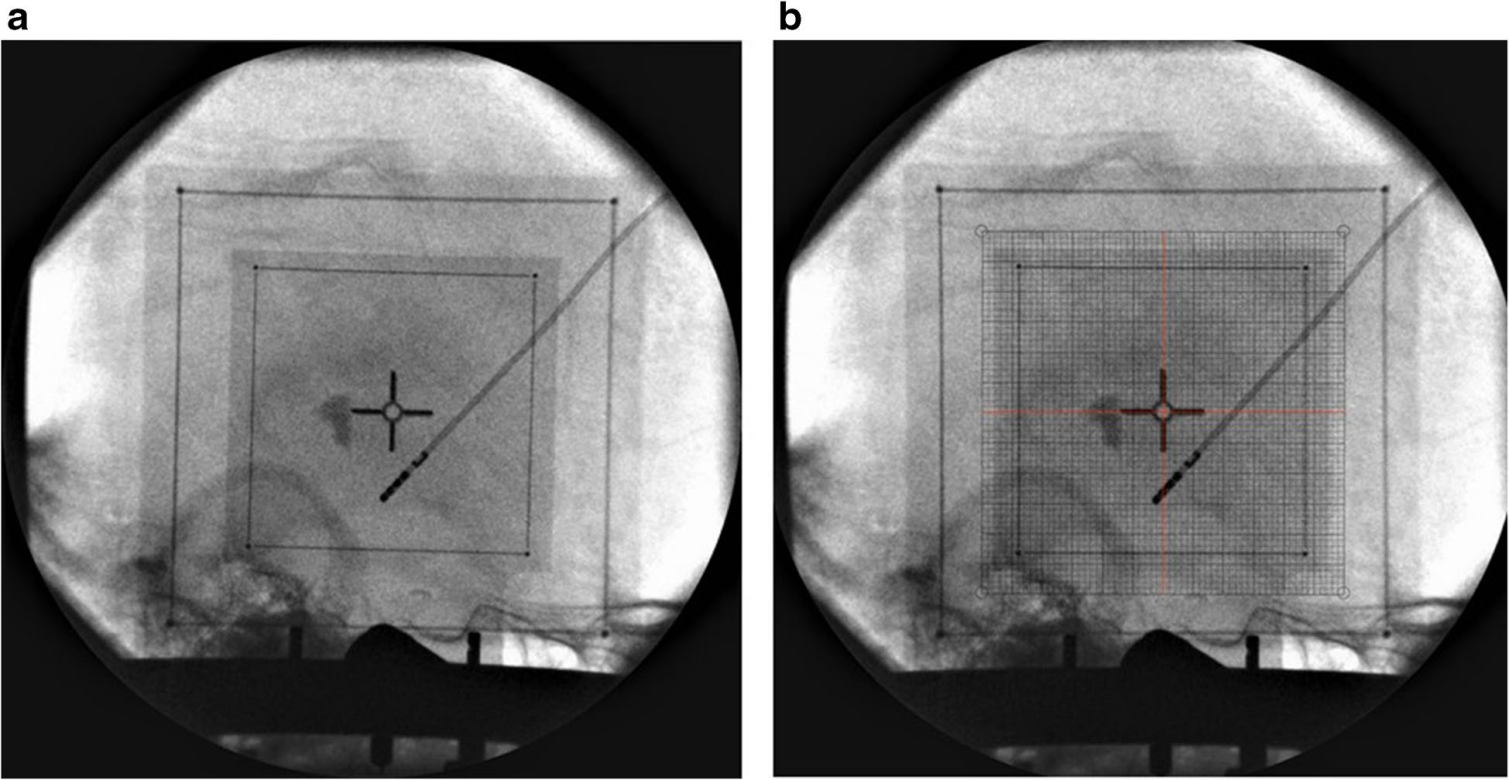
**a** Lateral view was obtained right after the first lead insertion. **b** A grid was printed and superimposed on the image. The coordinates were obtained (x NA, y −2, z + 46) and compared with planned coordinates

**Fig. 10:**
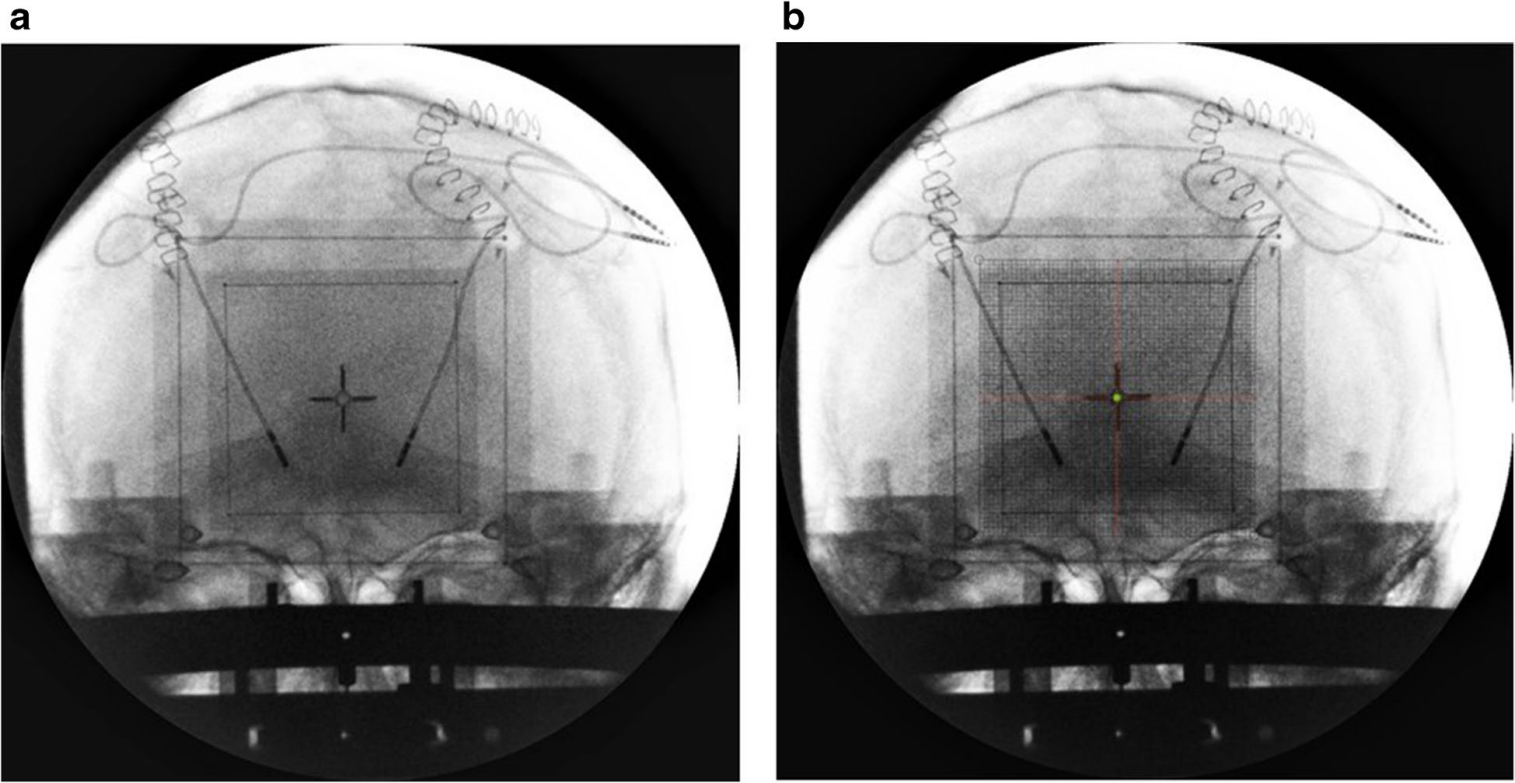
**a** After insertion of the other lead, the AP view was obtained to check laterality. **b** The grid was printed per the MF and superimposed over the image. The green dot was drawn to point to the center of the system (× 0, y NA, z + 60)

This technique can be used in both views, AP and lateral (Figs. 9 and 10). This imaging technique was tested intraoperatively. Images in both views were obtained during various stereotactic procedures and were compared with images obtained from our classical stereotactic fixedly-mounted X-ray system.

## Discussion/Conclusion

A fundamental prerequisite to our method is that the C-Arm is static throughout the procedure. The use of an isocentric C-Arm facilitates the shift between lateral and AP views without affecting the magnification factor. When repositioning the C-arm, it is essential to verify the accuracy of both the lateral and anteroposterior (AP) projections afterward and to make any necessary corrections.

To ensure an image-free divergence and thus exclude image distortion, our calculated imaginary plate should reside median in position to the group of stereotactic targets along the X-ray beam. This translates to the mid-sagittal plane when obtaining lateral view images for targeting thalamic and peri-thalamic structures. For functionally (relating to AC-PC line) extreme lateral targets, the magnification should be linearly adapted based on the distance between the target structure and the modified center of our stereotactic field (x = 0, y = 0, z = 60). This assumption however is a rare occurring necessity in applied stereotaxy. To calculate the magnification factor at any defined point along the X-ray beam, we concluded a unified linear Interpolation magnification formula that could predict the magnification factor at any point along the path between the projected localizer plates.

$$M_{plane}=M_1+\frac d{360}\times{(M}_1-M_2)$$

where

d: is the distance from the proximal plate to the plane of interest.
$$M_1$$ and $$M_2$$
are the magnifications of the projected proximal and distal plates on the image.

We have validated this technique under non-clinical conditions utilizing the target point simulator (stereotactic phantom) and with intraoperative images obtained during routine stereotactic procedures. The latter were acquired using our classical stereotactic fixedly-mounted X-ray system. We found identical results, with a mean difference of lower than 1 mm.

This simple geometrical adaptation proved to be an accurate, accessible, mobile, and manageable technique, providing immediate access to stereotactic coordinates during surgery. The accuracy proved to be non-inferior to other more complex and time-consuming imaging modalities.

## Data Availability

No datasets were generated or analysed during the current study.
